# Protective role of autophagy in mouse cecal ligation and puncture-induced sepsis model

**DOI:** 10.1186/cc11939

**Published:** 2013-03-19

**Authors:** W Takahashi, H Hatano, H Hirasawa, S Oda

**Affiliations:** 1 Graduate School of Medicine, Chiba University, Chiba, Japan; 2 Biomedical Research Center, Chiba University, Chiba, Japan

## Introduction

Autophagy is well known as one of the biogenic responses against various stresses, which possesses the beneficial roles for survival, but little is known about the dynamics and its significance during the septic condition. We hypothesized that autophagy is induced during the septic condition, and contributes to protect from tissue damage which subsequently leads to organ dysfunction. We confirm whether the autophagic process is accelerated or sustained in an acute phase of sepsis and we also determine its physiological role.

## Methods

Sepsis was induced by cecal ligation and puncture (CLP) in mice. We examined the kinetics of autophagosome and autolysosome formation which may explain the status of autophagy by western blotting, immunohistochemistry, and electron microscopy. To investigate a precise role of autophagy in CLP-induced sepsis, chloroquine, an autophagy inhibitor, was administered to the CLP-operated mice, and blood chemistry, pathology of the liver and survival were evaluated.

## Results

Autophagy demonstrated by the ratio of LC3-II/LC3-I was induced over the time course up to 24 hours after CLP. The ratio was particularly increased in the liver, heart and spleen. Autophagosome formation became maximal at 6 hours and declined by 24 hours after CLP. Autolysosome formation as evaluated by both fusion of GFP-LC3 dots with LAMP1 immunohistochemistry and electron microscopy was also increased after the procedure. Furthermore, inhibition of autophagy by chloroquine during the CLP procedure resulted in elevation of serum AST levels, and significantly increased mortality in mice.

## Conclusion

Autophagy was induced in several organs over the time course of the CLP sepsis model and then the process was gradually completed to degradation of the components. Our data suggest autophagy plays a protective role in organ dysfunction in sepsis.

